# The Kurtz–Perry Powder Technique Revisited: A Study of the Effect of Reference Selection on Powder Second-Harmonic Generation Response

**DOI:** 10.3390/molecules28031116

**Published:** 2023-01-22

**Authors:** Mengran Sun, Guili Wang, Jiyong Yao

**Affiliations:** 1 Beijing Center for Crystal Research and Development, Key Lab of Functional Crystals and Laser Technology, Technical Institute of Physics and Chemistry, Chinese Academy of Sciences, Beijing 100190, China; 2 Center of Materials Science and Optoelectronics Engineering, University of Chinese Academy of Sciences, Beijing 100049, China

**Keywords:** Kurtz–Perry technique, second-harmonic generation, light scattering effect, reference selection

## Abstract

The accurate evaluation of nonlinear optical (NLO) coefficient, the main parameter affecting light conversion efficiency, plays a crucial role in the development of NLO materials. The Kurtz–Perry powder technique can evaluate second-harmonic generation (SHG) intensity in pristine powder form, saving a significant amount of time and energy in the preliminary screening of materials. However, the Kurtz–Perry method has recently been subject to some controversy due to the limitations of the Kurtz–Perry theory and the oversimplified experimental operation. Therefore, it is very meaningful to revisit and develop the Kurtz–Perry technique. In this work, on the basis of introducing the light scattering effect into the original Kurtz–Perry theory, the theoretical expression of second-harmonic generation intensity with respect to band gap and refractive index are analyzed. In addition, the reference-dependent SHG measurements were carried out on polycrystalline LiB_3_O_5_ (LBO), AgGaQ_2_ (Q = S, Se), BaGa_4_Q_7_ (Q = S, Se), and ZnGeP_2_ (ZGP), and the results of SHG response emphasize the importance of using appropriate references to the Kurtz–Perry method. In order to obtain reliable values of nonlinear coefficients, two criteria for selecting a reference compound were proposed: (1) it should possess a band gap close to that of the sample to be measured and (2) it should possess a refractive index close to that of the sample to be measured. This work might shed light on improvements in accuracy that can be made for effective NLO coefficients obtained using the Kurtz–Perry method.

## 1. Introduction

As the core component of all solid-state lasers, nonlinear optical (NLO) crystals can obtain the desired laser frequency by using NLO technologies such as sum frequency generation (SFG), difference frequency generation (DFG), and optical parametric oscillation (OPO) [1,2,3,4,5,6,7]. At present, NLO materials have been widely used in military and civil fields, including the development of laser radar, laser ranging, environmental monitoring, medical treatment, medical diagnosis, etc. [8,9,10,11,12,13,14,15]. The key parameter affecting optical conversion efficiency is the second-order NLO polarizability, χ^(2)^, of crystals [16,17]. Generally speaking, accurate NLO coefficients can be obtained by using Maker fringes and derivation techniques [18,19,20]. However, in the above experiments, large-size single crystals need to be grown, oriented, cut, and polished to obtain full NLO tensors. This process is very time-consuming and not suitable for material preliminary screening. In addition, this method cannot be used when single crystals cannot be obtained. Therefore, in the process of preliminary exploration of materials, the Kurtz–Perry powder technique has gradually become a general method for the evaluation of the NLO response and phase-matching (PM) characteristics of materials [2,12,21,22,23,24,25,26,27].

The Kurtz–Perry powder technique was first proposed by S. K. Kurtz and T. T. Perry in 1968 [21]. This technique utilizes a dual-beam oscilloscope, which depends on the measurement of a specimen relative to a reference compound with known NLO coefficients [28]. The dominant advantage of Kurtz–Perry technology is that the approximate NLO coefficients of compounds can be obtained by using its powder form, which greatly improves screening efficiency [21]. More importantly, the types of compounds that can be measured using the Kurtz–Perry method are broad (including chalcogenides, halides, oxides, borates, carbonates, hydroxides, iodates, etc.), indicating the versatility of the method [29,30,31,32,33,34,35,36,37]. However, the Kurtz–Perry method has recently been re-analyzed by scientists. Some of them suggest that the Kurtz–Perry technique might give rise to some errors when evaluating the NLO coefficient of materials, especially in narrow band gap systems, as it fails to consider the effect of light scattering in the second-harmonic generation (SHG) measurement process [38]. Moreover, the Kurtz–Perry technique does not consider the packing fraction of powder theoretically, which may lead to misleading results [39]. Thus, the light scattering effect was introduced into the original Kurtz–Perry model by Aramburu et al., and its influence on SHG intensity was theoretically analyzed [38]. Moreover, several alternative methods to the Kurtz–Perry technique have also been developed to compensate for the shortcomings, such as SHG measurements performed by an evanescent wave under total reflection conditions or the determination of d_ij_ using powder crystal monolayers [40,41]. Undoubtedly, these revisions have improved the accuracy of the Kurtz–Perry method to a certain degree, but they did not consider the impact of the selected reference on the conclusions obtained from measurement and data processing.

Recently, Clark et al. demonstrated that the use of commercial and homemade grades of the same polycrystalline reference can result in significant deviations in the NLO coefficients of LiInSe_2_ [28]. However, the possible influence of using different compounds as standard on SHG measurement results has not been discussed yet. It can be speculated that a significant influence on the experimental conclusion will be caused by an inappropriate reference, as the Kurtz–Perry technique is a semi-quantitative method that predicts an approximation of the effective NLO coefficient of the sample based on the SHG intensity of the reference material. Therefore, the selection of a matching reference material is of great significance to NLO performance prediction. Materials currently available to be used as a reference include but are not limited to β-BaB_2_O_4_ (β-BBO), KH_2_PO_4_ (KDP), LiB_3_O_5_ (LBO), KTiOPO_4_ (KTP), AgGaS_2_ (AGS), AgGaSe_2_ (AGSe), and ZnGeP_2_ (ZGP) [42,43,44,45,46,47]. In the above-mentioned compounds, BBO, KDP, LBO, and KTP can be widely used in the ultraviolet-vis-near-infrared regions, while AGS, AGSe, and ZGP are widely used in *mid*- and *far*-infrared regions. The linear and nonlinear optical properties of these crystals are summarized in Table 1. Obviously, these compounds exhibit various optical properties, including band gap, refractive index, effective NLO coefficient, and transmission range. Therefore, how to screen out the appropriate benchmark material according to different systems is an important aspect to consider when seeking to improve the accuracy of the Kurtz–Perry method. In this work, we explored the differences in SHG measurement coefficients caused by using several different references under the same measurement conditions, namely AGQ (Q = S, Se), BaGa_4_S_7_ (BGS), BaGa_4_Se_7_ (BGSe), ZGP, and LBO. In addition, on the basis of introducing the light scattering effect into the original Kurtz–Perry theory, the theoretical expressions of band gap and refractive index on SHG intensity were derived, and two criteria for selecting appropriate reference materials have been proposed.

## 2. Results and Discussion

### 2.1. Experimental Results

**Figure 1:**
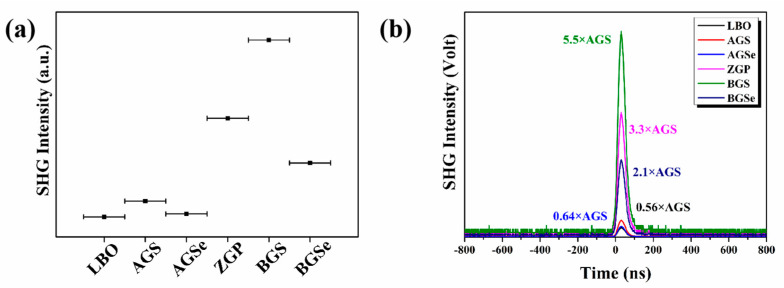
(**a**) The SHG intensities of LBO, AGQ (Q = S, Se), ZGP, and BGQ (Q = S, Se) samples at particle sizes of 20−50 μm; (**b**) oscilloscope traces of SHG signals for LBO, AGQ (Q = S, Se), ZGP, and BGQ (Q = S, Se) samples (AGS as a reference).

The powder SHG responses of LBO, AGQ (Q = S, Se), ZGP, and BGQ (Q = S, Se) with particle sizes of 20–50 μm were explored using a 2090 nm laser as the fundamental light, and the results are illustrated in Figure 1a. The SHG intensities of the six compounds are ranked in the following order: BGS, ZGP, BGSe, AGS, AGSe, and LBO. Interestingly, BGS has the strongest SHG response (although its NLO coefficient is not the largest), while LBO and AGSe have similar SHG response strength (although the NLO coefficient of AGS is about 21 times that of LBO). Moreover, taking AGS as the reference, the SHG intensities of the other five substances relative to the reference were obtained (Figure 1b). The d_eff_ values of these five compounds were then determined one by one through Equation (15). It is worth noting that the effective NLO coefficients obtained by using AGS as a reference in this work are quite different from those reported in the literature. The other five substances were then selected as the reference material, and the effective NLO coefficients of these materials were also obtained. The results of this work and those mentioned in the literature are summarized in Table 2, and there are significant differences between them.

### 2.2. Theoretical Analysis of Second-Harmonic Generation Intensity

Therefore, we consider that the reason for the above huge difference is the selection of mismatched reference materials. In general, the six substances mentioned above were usually selected as references because they are commercially available and are well known. Based on the above discussion, we believe that an appropriate reference material is necessary for obtaining accurate SHG coefficients using the Kurtz–Perry method. Thereby, what conditions should an appropriate reference satisfy? Herein, the SHG intensities measured by the Kurtz–Perry method for powder samples were theoretically analyzed.

**Figure 2:**
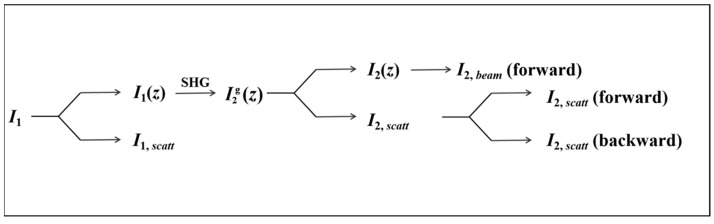
Second-harmonic generation (SHG) process diagram.

A SHG process diagram is depicted in Figure 2. As proposed by Aramburu et al., light scattering by the particles is explicitly introduced in the model [38]. According to the analysis in the paper published by Aramburu et al. in 2013, it is assumed that the sample can be modeled by a plate whose *x* and *y* dimensions are far greater than the sample thickness (*L*) and the main beam incident along the *z*-axis can be described by a plane wave. It is also assumed that the diameter of the incident light (*D*) is much greater than the thickness of the sample (*D >> L*). These assumptions simplify this problem to a one-dimensional case in the *z* direction. In addition, as the SHG intensity is proportional to $I_{1}^{2}$, the contribution of the expected scattering intensity to SHG is negligible, thus SHG response is dominantly generated by fundamental light *I*_1_*(z)*. The generated SHG beam $I_{g2}$(*z*) partially propagates along the *z* axis, and the SHG intensity passing through the sample surface at *z* = *L is I*_2,*beam*_, while the other part (I_2,scatt_) diffuses to the whole sample via the scattering effect of dry powder before finally passing through all its surfaces (forward and backward). Therefore, in the general case, the total SHG intensity emitted by the sample will be given by:

(1) $$I_{2,\;total}=I_{2,\;beam}\left(forward\right)+I_{2,\;scatt}$$

where

(2) $$I_{2,\;beam}\left(forward\right)=q\left(\frac{g\left(r\right)}{r}\right)\frac{l_{1}l_{2}}{\left(2l_{2}-l_{2}\right)}\left(exp\left(-\frac{L}{l_{2}}\right)-exp\left(-\frac{2L}{l_{1}}\right)\right)$$

(3) $$I_{2,\;scatt}=q\left(\frac{g\left(r\right)}{r}\right)\frac{l_{1}l_{2}}{\left(2l_{2}-l_{1}\right)}\left(1-exp\left(-\frac{L}{l_{2}}\right)-\frac{l_{1}}{2l_{2}}\left(1-exp\left(-\frac{2L}{l_{1}}\right)\right)\right)$$

where *r* represents the particle size and *l*_1_ and *l*_2_ are effective mean free path of fundamental and second-harmonic light, respectively.

Thus,

(4) $$q=\frac{3}{2}fT_{2}T_{1}^{2}I_{1,o}^{2}$$

(5) $$g\left(r\right)=\frac{8\Pi^{2}}{\varepsilon_{0}{c\lambda }_{1}}{\mathrm{PolFact}\;\mathrm{nFact}\;d}_{\mathrm{eff}}^{2}r$$

Here, *f* is the volume packing fraction, *T*_1_ represents the transmittance of fundamental light between medium and material, *T*_2_ represents the transmittance of second-harmonic light between medium and material, and *I*_1,0_ is intensity of fundamental light at *z* = 0. Moreover, *g*(*r*) is the function of particle size, second-order susceptibility coefficients *d_ij_*, and refractive indices; *ε_o_* represents the vacuum dielectric constant; *c* is the light speed; and *λ*_1_ is the wavelength of the fundamental wave. PolFact is the “polarization factor” and nFact is a function of the principal refractive indices of the material for the wavelengths involved {(n_1o_, n_1e_), (n_2o_, n_2e_)}. This depends on the sign of the birefringence and the type of PM. The values of PolFact and nFact are summarized in Table 3. Thus, the expression of *I*_total_ can be rewritten as:

(6) $$I_{2,\;total\;}=q\left(\frac{g\left(r\right)}{r}\right)\frac{l_{1}}{2}\left(1-exp\left(-\frac{2L}{l_{1}}\right)\right)$$

Since the sample is dry powder, the effective mean free path *l*_1_ is usually small. Assuming that the sample is thick enough, that is, $\frac{l_{1}}{2}$ << *L*, SHG strength is approximately independent of the sample thickness *L*:

(7) $$I_{2,\;total\;}=\underset{\frac{2L}{l_{1}}\to \infty }{\lim}q\left(\frac{g\left(r\right)}{r}\right)\frac{l_{1}}{2}\left(1-exp\left(-\frac{2L}{l_{1}}\right)\right)=q\left(\frac{g\left(r\right)}{r}\right)\frac{l_{1}}{2}$$

where

(8) $$l_{1}\approx \frac{2r}{3f}=l_{2}$$

Substituting Equations (5) and (8) into Equation (7), analytical expressions for *I*_2,total_ can be obtained:

(9) $$I_{2,\;total}≌\frac{1}{2}T_{2}T_{1}^{2}I_{1,o}^{2}g\left(r\right)$$

Equations (1)–(9) were proposed by Aramburu et al. in 2013 [38]. On this basis, we carried out a further discussion. According to the calculation of absorption coefficient, the transmission expression can be given by [59]:

(10) $$T=\frac{-e^{\alpha L}{\left(1-R\right)}^{2}}{R^{2}-e^{2\alpha L}}$$

*α* is the absorption coefficient [60,61]:

(11) $$\alpha =\frac{{\left[A\left(h\upsilon -E_{g}\right)\right]}^{\frac{1}{m}}}{h\upsilon }$$

where $m=\{\begin{array}{c} 2,\;direct\;bandgap\\ 1/2,\;indirect\;bandgap \end{array}$, *R* is reflection coefficient, and

(12) $$R=\frac{{\left(n-1\right)}^{2}}{{\left(n+1\right)}^{2}}$$

where n is refractive index.

Substituting Equations (11) and (12) into Equation (10), the transmission expression can be given by:

(13) $$T=\frac{-16n^{2}\exp\left(\frac{{\left(A\left(h\upsilon -E_{g}\right)\right)}^{\frac{L}{m}}}{h\upsilon }\right)}{{\left(n-1\right)}^{4}-{\left(n+1\right)}^{4}\exp\left(\frac{{\left(A\left(h\upsilon -E_{g}\right)\right)}^{\frac{2L}{m}}}{h\upsilon }\right)}$$

*I*_2,total_ then becomes dependent on E_g_, n, d_eff_, and r of the sample:

$$I_{2,\;total}=\left[\frac{-16n_{2}^{2}\exp\left(\frac{{\left(A\left(h\upsilon -E_{g}\right)\right)}^{\frac{L}{m}}}{h\upsilon }\right)}{{\left(n_{2}-1\right)}^{4}-{\left(n_{2}+1\right)}^{4}\exp\left(\frac{{\left(A\left(h\upsilon -E_{g}\right)\right)}^{\frac{2L}{m}}}{h\upsilon }\right)}\right]{\left[\frac{-16n_{1}^{2}\exp\left(\frac{{\left(A\left(h\upsilon -E_{g}\right)\right)}^{\frac{L}{m}}}{h\upsilon }\right)}{{\left(n_{1}-1\right)}^{4}-{\left(n_{1}+1\right)}^{4}\exp\left(\frac{{\left(A\left(h\upsilon -E_{g}\right)\right)}^{\frac{2L}{m}}}{h\upsilon }\right)}\right]}^{2}$$

(14) $$\frac{4\Pi^{2}I_{1,o}^{2}\mathrm{nFact}}{\varepsilon_{0}{c\lambda }_{1}}d_{\mathrm{eff}}^{2}r$$

### 2.3. Two Criteria for Selecting Reference Materials

According to Equations (9) and (14), total SHG intensity mainly depended on the band gap (E_g_), refractive index (n), effective NLO coefficient (d_eff_), and particle size (r) of the material. Generally, in the process of SHG measurement, a standard sieve was used to divide the samples and the reference into the same particle size range. Notably, SHG intensity is only a function of d_eff_ when SHG measurements were performed under the same experimental conditions (i.e., r, I_0_, and λ_1_ are consistent) and the refractive index and band gap of the sample were similar to those of the reference material. This conclusion is consistent with Equation (15). In this case, a qualified reference material should satisfy the following two criteria: (1) it should have a band gap close to that of the sample to be measured, which can minimize the issue of SHG response attenuation caused by optical absorption; and (2) it should have a refractive index close to that of the sample to be measured, which can minimize SHG intensity reduction caused by light scattering effects.

Herein, taking biaxial crystals satisfying type-I phase matching as an example (therefore, $g\left(r\right)=\frac{3\pi^{2}}{\varepsilon_{0}c\lambda_{1}}d_{eff}^{2}r$), the effect of band gap and refractive index on SHG intensity is qualitatively analyzed. Firstly, the effect of band gap on SHG intensity is discussed. According to Equation (11), the smaller E_g_ is, the stronger the absorption α of the compound to the fundamental frequency light and the second-harmonic light. According to Equation (10), the transmittance of the compound to the fundamental frequency light and the second harmonic light decreases with the increase in absorption α. Finally, according to Equation (9), the total SHG intensity measured by the Kurtz–Perry method decreases with the decrease in T_1_ and T_2_. In particular, when the wavelength corresponding to the band gap of a compound is close to 1045 nm, the experimental SHG intensity will be much lower than the theoretical value. For example, the band gap of Ba_2_Ge_2_Te_5_ is 1.15 eV, and the measured SHG intensity is about 0.45 times that of AGS while the theoretical NLO coefficient is about 16 times that of AGS. Secondly, the effect of refractive index on SHG intensity is also analyzed. On the one hand, according to Equation (13), both the numerator and the denominator of T decrease as the refractive index n increases, but the rate of decrease in the numerator is greater than that in the denominator. Thus, T decreases as n increases. On the other hand, the increased refractive index of the compound leads to an enhanced scattering effect, which reduces the SHG intensity that can be collected. Based on the above discussion and analysis, SHG intensity measured by the Kurtz–Perry method is significantly affected by the band gap and refractive index. The crystallographic information and linear optical properties of LBO, AGQ (Q = S, Se), ZGP, and BGQ (Q = S, Se) are listed in Table 1. Taking LBO and AGS as an example, the band gap of LBO is largest (about 7.37 eV). On the one hand, its wide band gap is conducive to reduced absorption of the generated 1 μm frequency-doubled light. On the other hand, its low refractive index also makes it have a weak scattering effect. As a result, the SHG intensity of LBO obtained with a 2.09 μm laser is approximately 0.6 times that of AGS, although its effective NLO coefficient is only 1/20.9 times that of AGS. In addition, the SHG intensities of AGS and AGSe were 2.23 and 1.43, respectively, although the effective NLO coefficient of AGSe is 2.1 times that of AGS. Undoubtedly, the crystallization quality of the sample will affect the strength of SHG to some extent [28]. However, we propose the following considerations: Firstly, the band gap of AGS is significantly wider than that of AGSe, and the narrow band gap of AGSe results in strong absorption and low transmission of SHG waves, that is, *I*_2,beam_ decreases. Secondly, the larger refractive index of AGSe leads to the SHG wave being diffused throughout the sample via a scattering effect, and the contribution of *I*_2,scatt_ is also reduced. Therefore, the measured SHG signal of AGSe is weaker than that of AGS.

In these cases, selecting a reference which exhibits similar linear optical properties to the sample to be measured is of great significance. Taking BGSe as an example and selecting AGS or AGSe as the reference, the NLO coefficient calculated according to Equation (15) with AGS as the reference is 19.24 pm/V, while the coefficient obtained with AGSe as the reference is 50.75 pm/V. Obviously, the result obtained by the former is closer to the actual value. The reason for this phenomenon is that the band gap and refractive index of AGS are closer to the band gap and refractive index of BGSe than of AGSe (Table 1). We believe that experimental studies using the Kurtz–Perry technique when evaluating NLO performance will become more convincing through consideration of the above two principles in the selection of references.

## 3. Experimental Section

### 3.1. Experimental Apparatus

**Figure 3:**
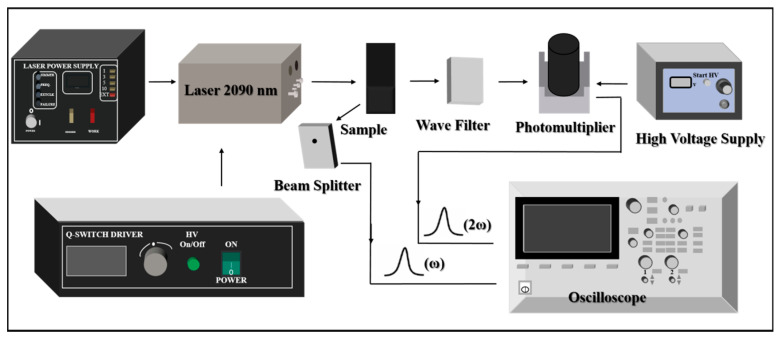
Apparatus utilized for measurement of second-harmonic generation in powders.

The powder second-harmonic generation (SHG) configuration is detailed in Figure 3. A 2090 nm laser generated by Q-switched Ho:Tm:Cr:YAG was used as the fundamental light. When the pulse laser emitted by the laser passes through the powder sample, the signal of the scattered fundamental frequency light is detected by the beam splitter and transmitted to the oscilloscope for display. A part of the fundamental frequency light will generate the signal of the second-harmonic light through the sample to be measured. After the fundamental frequency light is filtered by the wave filter, the generated second-harmonic light will be gathered on the photomultiplier for amplification, and the signal intensity on the photomultiplier will then be converted into the second-harmonic light electrical signal, which will be displayed and recorded by the oscilloscope. The oscilloscope displays the fundamental frequency signal and the frequency-doubled light signal at the same time to present the intensities of the fundamental wave and the second-harmonic light in the sample.

### 3.2. Sample Preparation

For this quantitative work, AGQ (Q = S, Se), BGQ (Q = S, Se), ZGP, and LBO powder samples were graded using a standard sieve with a particle size range of 20–50 μm. These samples were then clamped between two glass microscope slides (a 0.5 mm thick rubber gasket was added between the glass slides to ensure consistent sample thicknesses) and secured with tape in a 1 mm thick aluminum holder.

### 3.3. Second-Harmonic Generation Measurement

All NLO measurements were performed at room temperature. The measurement device is shown in Figure 3. Laser pulse duration and pulse repetition frequency for SHG measurements were 100 ns and 1 HZ, respectively. The magnitude of the frequency doubling effect is determined by comparing the electrical signal strength of the sample with that of the reference. The second-order NLO coefficient *d_eff_(S)* of the sample is calculated by the following formula [28]:

(15) $$d_{eff}\left(S\right)=d_{eff}\left(R\right){\left(\frac{I_{S}\left(2\omega \right)}{I_{R}\left(2\omega \right)}\right)}^{\frac{1}{2}}$$

where *I_S_(2ω)* and *I_R_(2ω)* represent the SHG intensities of the sample to be measured and the reference material, respectively. Additionally, *d_eff_(R)* is the effective NLO coefficient of the reference.

## 4. Conclusions

The Kurtz–Perry method saves time and energy in material preliminary screening, but it also has some limitations. Therefore, optimizing the Kurtz–Perry theory for subsequent material evaluation is of great significance. Herein, on the basis of introducing the light scattering effect into the Kurtz–Perry theory, the theoretical expressions of band gap and refractive index on SHG intensity are derived. Subsequently, two factors affecting SHG intensity were deduced through theoretical analysis: band gap and refractive index. By comparison, the qualified reference should meet the following two standards: (1) it should possess a band gap close to that of the sample to be measured, which can minimize SHG response attenuation caused by light absorption; and (2) it should possess a refractive index close to that of the sample to be measured, which can minimize the influence of light scattering effects. This work may give rise to new inspiration of SHG evaluation in the future.

## Figures and Tables

**Table 1:** Crystallographic information and linear and nonlinear optical properties of some known crystals.

Compound	S. G.	E_g_ (eV)	Refractive Index@1045 nm	Transmission Range (μm)	d_eff_ (pm/V) ^a^	Reference
LBO	*Pna*2_1_	7.37	n_x_ = 1.5651 n_y_ = 1.5908 n_z_ = 1.6057	0.60–2.6	d_eff_ = 0.64	[3]
β-BBO	*R*3	6.53	n_o_ = 1.6546 n_e_ = 1.5393	0.189–3.5	d_eff_ = 2.01	[20,48]
KDP	*I*-42*d*	6.97	n_o_ = 1.4944 n_e_ = 1.4601	0.18–1.7	d_eff_ = 0.26	[48,49]
KTP	*Pna*2_1_	3.52	n_x_ = 1.7391 n_y_ = 1.7464 n_z_ = 1.7902	0.35–4.5	d_eff_ = 3.58	[48,50]
AGS	*I*-42*d*	2.75	n_o_ = 2.4549 n_e_ = 2.4021	0.50–13	d_eff_ = 13.4	[51]
AGSe	*I*-42*d*	1.82	n_o_ = 2.7044 n_e_ = 2.6838	0.73–17	d_eff_ = 28.3	[52]
ZGP	*I*-42*d*	1.75	n_o_ = 3.2759 n_e_ = 3.3313	0.70–12	d_eff_ = 34.3	[52,53]
BGS	*Pmn*2_1_	3.59	n_x_ = 2.2828 n_y_ = 2.3024 n_z_ = 2.3231	0.54–9.4	d_eff_ = 5.38	[54,55]
BGSe	*Pc*	2.73	n_x_ = 2.4897 n_y_ = 2.5047 n_z_ = 2.5641	0.70–14.8	d_eff_ = 14.7	[54,56]

^a^ the effective nonlinear coefficient d_eff_ was calculated using the SNLO program [57,58].

**Table 2:** Powder SHG intensity and the calculated SHG coefficient from different reference materials.

Compound	SHG Intensity	d_eff_ (LBO as Reference) ^b^	d_eff_(AGS as Reference) ^b^	d_eff_ (AGSe as Reference) ^b^	d_eff_ (ZGP as Reference) ^b^	d_eff_ (BGS as Reference) ^b^	d_eff_ (BGSe as Reference) ^b^	d_eff_ (pm/V) ^a^
LBO	1.25	0.64	10.03	26.46	14.1	1.72	7.66	0.64
AGS	2.23	0.85	13.4	35.23	18.79	2.29	10.23	13.4
AGSe	1.43	0.68	10.74	28.3	15.09	1.83	8.18	28.3
ZGP	7.39	1.56	24.4	64.33	34.3	4.17	25.91	34.3
BGS	12.25	2.80	31.4	82.83	44.16	5.38	18.63	5.38
BGSe	4.60	1.23	19.24	50.75	27.06	3.30	14.7	14.7

^b^ this work.

**Table 3:** PolFact and nFact for each type of PM.

Optical Property	Positive/Negative	Type of PM	PolFact	nFact
uniaxial	positive: n_e_ > n_o_	I	3/8	$\frac{1}{2}\frac{n_{1o}^{2}n_{1e}^{2}}{cos\left(\theta_{c}\right)n_{2o}^{6}\left(n_{1e}^{2}-n_{1o}^{2}\right)}$
		II	1/2	$\frac{n_{1o}n_{1e}^{2}}{cos\left(\theta_{c}\right)n_{2o}{\left(2n_{2o}-n_{1o}\right)}^{4}\left(n_{1e}^{2}-n_{1o}^{2}\right)}$
	negative: n_e_ < n_o_	I	3/8	$\frac{1}{2}\frac{n_{2o}^{2}n_{2e}^{2}}{cos\left(\theta_{c}\right)n_{1o}^{6}\left(n_{2o}^{2}-n_{2e}^{2}\right)}$
		II	1/2	$\frac{1}{cos\left(\theta_{c}\right)n_{2e}\left(\theta_{c}\right)n_{1o}n_{1e}\left(\theta_{c}\right)\left[2n_{2e}^{3}\left(\theta_{c}\right)\left(\frac{1}{n_{2e}^{2}-n_{2o}^{2}}\right)-n_{1e}^{3}\left(\theta_{c}\right)\left(\frac{1}{n_{1e}^{2}-n_{1o}^{2}}\right)\right]}$
biaxial	positive: n_e_ > n_o_	I	3/8	1
		II	1/2	1
	negative: n_e_ < n_o_	I	3/8	1
		II	1/2	1

θ_c_ is the angle of phase matching.

## Data Availability

The data will be made available by the corresponding author on reasonable request.
